# Measuring health literacy in populations: illuminating the design and development process of the European Health Literacy Survey Questionnaire (HLS-EU-Q)

**DOI:** 10.1186/1471-2458-13-948

**Published:** 2013-10-10

**Authors:** Kristine Sørensen, Stephan Van den Broucke, Jürgen M Pelikan, James Fullam, Gerardine Doyle, Zofia Slonska, Barbara Kondilis, Vivian Stoffels, Richard H Osborne, Helmut Brand

**Affiliations:** 1 Department of International Health, CAPHRI, Faculty of Health, Medicine and Life Sciences, Maastricht University, P.O. Box 616, 6200, Maastricht, MD, the Netherlands; 2 Department of Psychology, Catholic University of Louvain, Leuven, Belgium; 3 Ludwig Boltzmann Institute Health Promotion Research, Vienna, Austria; 4 School of Public Health, Physiotherapy and Population Science and School of Business, University College Dublin, Dublin, Ireland; 5 School of Business, University College Dublin, Dublin, Ireland; 6 The Cardinal Wyszyński Institute of Cardiology, Warsaw, Poland; 7 Hellenic American University, Manchester, NH, USA; 8 Hellenic American Education Center, Athens, Greece; 9 Public Health Innovation, Deakin Population Health Strategic Research Centre, Deakin University, VIC 3125, Melbourne, Australia

**Keywords:** Health literacy, Survey, Measurement, Tool, Population

## Abstract

**Background:**

Several measurement tools have been developed to measure health literacy. The tools vary in their approach and design, but few have focused on comprehensive health literacy in populations. This paper describes the design and development of the European Health Literacy Survey Questionnaire (HLS-EU-Q), an innovative, comprehensive tool to measure health literacy in populations.

**Methods:**

Based on a conceptual model and definition, the process involved item development, pre-testing, field-testing, external consultation, plain language check, and translation from English to Bulgarian, Dutch, German, Greek, Polish, and Spanish.

**Results:**

The development process resulted in the HLS-EU-Q, which entailed two sections, a core health literacy section and a section on determinants and outcomes associated to health literacy. The health literacy section included 47 items addressing self-reported difficulties in accessing, understanding, appraising and applying information in tasks concerning decisions making in healthcare, disease prevention, and health promotion. The second section included items related to, health behaviour, health status, health service use, community participation, socio-demographic and socio-economic factors.

**Conclusions:**

By illuminating the detailed steps in the design and development process of the HLS-EU-Q, it is the aim to provide a deeper understanding of its purpose, its capability and its limitations for others using the tool. By stimulating a wide application it is the vision that HLS-EU-Q will be validated in more countries to enhance the understanding of health literacy in different populations.

## Background

Health literacy is a composite term used to describe the capacities of persons to meet the complex demands related to health in modern society. As an outcome of health education and communication activities, it represents the cognitive and social skills that determine the motivation and ability of individuals to gain access to, understand and use information in ways that promote and maintain good health [1]. The concept has gained increasing attention both in research and practice due to its close association to the social determinants of health [2], health behavior and health outcomes [3], health service use [4] and quality of health systems as well as capacity building for professionals [5]. Along with the increasing interest in empirical work on health literacy, there has been a growing demand for tools to measure health literacy [6].

The existing tools that purport to measure health literacy vary in their approach and design, as well as in terms of their purpose. Some tools have been developed for the purpose of screening, and serve to divide people into categories with low or high levels of health literacy. Examples of this kind of tool are the Rapid Estimate of Adult Literacy in Medicine (REALM) [7,8], the Test of Functional Health Literacy (TOFHLA) [9-11] and the Newest Vital Sign (NVS) [12]. As they are often used in clinical settings, these tools are necessarily short and quick and easy to use. Other tools aim at measuring a broader concept of health literacy, with a view to provide an in-depth assessment of the dimensions of health literacy, or to explore its relationships with social determinants, health behavior, health status or healthy service use such as the National Assessment of Adult Literacy survey (NAAL) [13], the Critical Health Competence Test (CHC) [14], the Swiss Health Literacy Survey [15], the Health Literacy Management Scale (HeLMS) [16] and the Health Literacy Questionnaire (HLQ) [17]. Furthermore, existing health literacy measurement tools differ in terms of their administration style and their focus on specific aspects, such as the recognition and pronunciation of medical terms, numeracy, comprehension, and decision-making competencies. In terms of their technical qualities, the tools differ in terms of scoring and ranges. Accordingly, the time and resources needed for application also vary [18,19]. Yet, in spite of the wide range of tools that are available, it is recognized that many have substantial weaknesses [20]. Existing tools are far from optimal and show several limitations [21]. The most apparent shortcomings of most tools are that they fail to capture all relevant aspects of health literacy and only focus on one or a few dimensions of the concept; that they have a primary focus on personal attributes at the cost of population aspects; that they have an unclear relationship to current definitions and conceptual frameworks of health literacy; and that they show only weak associations with causes and outcomes of health literacy [20]. According to Pleasant et al. [6], a comprehensive measure of health literacy should reflect the following attributes: build explicitly on a testable theory or conceptual framework of health literacy; be multi-dimensional in content and methodology, to reflect the emerging theories of health literacy as a construct with multiple conceptual domains and practical components; use multiple methods; distinguish health literacy clearly from communication; treat health literacy as a latent construct, in the sense that the measure should include multiple items that sample from the conceptual domains outlined by the underlying theory or conceptual framework; honor the principle of compatibility in the sense that the measure should not focus exclusively on the clinical setting to research public health behaviors and outcomes; allow comparison and/or be commensurate across contexts including culture, life course, population group, and research setting; and prioritize social research and public health applications versus clinical screening [6].

To accomplish the European Health Literacy Survey (HLS-EU), which aimed to measure and compare health literacy in populations in selected countries in Europe [22], the HLS-EU Consortium consisting of nine research institutes from Austria, Bulgaria, Germany^a^, Greece, Ireland, the Netherlands, Poland and Spain, developed the European Health Literacy Survey Questionnaire (HLS-EU-Q). It embraces the principles outlined by Pleasant and colleagues and captures the essential dimensions of health literacy as outlined in the definition and conceptual model proposed by Sorensen et al. [23].

The present paper describes the process of developing the HLS-EU-Q. Specifically, it provides a detailed outline of the structured and systematic approach that was taken concerning the item generation, pre-testing, field-testing, external consultation, plain language check, and translation of the tool, with the purpose of creating and testing a concept based, multidimensional, multinational, interdisciplinary and comprehensive measurement of health literacy in populations. As such, the paper provides insight to the extensive development process of designing the HLS-EU-Q, which is useful for its subsequent application and validation. Initially, the methods are described for each step performed in the development process. Then the results for each step are presented. Finally the development process and the attributes of the HLS-EU-Q are discussed in terms of quality and limitations.

## Method

### Applying a concept validation approach

In line with the principles outlined by Pleasant et al. [6], the development of the HLS-EU-Q followed a concept validation approach. Therefore, the design process was guided by the conceptual model of health literacy derived from a systematic literature review of existing definitions and conceptualisations of the concept by Sorensen et al. [23]. The model starts from a definition of health literacy which integrates the different aspects of health literacy as identified in the literature, stating that:

*“Health literacy is linked to literacy and entails the motivation, knowledge and competencies to access, understand, appraise and apply health information in order to make judgements and take decisions in everyday life concerning healthcare, disease prevention and health promotion to maintain or improve quality of life throughout the course of life”*[23].

Based on this definition, the HLS-EU Consortium developed a conceptual framework outlining the main dimensions of health literacy as mentioned in the literature, and integrating them in a logical model identifying the proximal and distal factors that may impact on health literacy, as well as potential consequences of health literacy in terms of health related behaviors, health outcomes and health service use [23]. The core of the model can be conceived of as a 12 cell matrix positing the key processes off accessing, understanding appraising and applying health-related information within three domains (Table 1) [23]:

(1) The domain of *healthcare*, where health literacy refers to the ability to access information on medical or clinical issues, to understand medical information, to interpret and evaluate medical information, and to make informed decisions on medical issues and comply with medical advice.

(2) The domain of *disease prevention*, where health literacy involves the ability to access information on risk factors for health, to understand information on risk factors and derive meaning, to interpret and evaluate information about risk factors, and to make informed decisions to protecting against risk factors for health.

(3) The domain of *health promotion*, where health literacy refers to the ability to regularly update oneself on determinants of health in the social and physical environment and derive meaning, to interpret and evaluate information on determinants of health in the social and physical environment, and the ability to make informed decisions on health determinants in the social and physical environment and also engage in joint action.

**Table 1:** HLS-EU Health literacy matrix

	**Access/obtain information relevant to health**	**Understand information relevant to health**	**Process/appraise information relevant to health**	**Apply/use information relevant to health**
**Healthcare**	Ability to access information on medical and clinical issues	Ability to understand medical information and derive meaning	Ability to interpret and evaluate medical information	Ability to make informed decisions on medical issues
**Disease prevention**	Ability to access information on risk factors for health	Ability to understand information on risk factors and derive meaning	Ability to interpret and evaluate information on risk factors for health	Ability to make informed decisions on risk factors for health
**Health promotion**	Ability to update oneself on determinants of health in the social and physical environment	Ability to understand information on determinants of health in the social and physical environment and derive meaning	Ability to interpret and evaluate information on health determinants in the social and physical environment	Ability to make informed decisions on health determinants in the social and physical environment

### Questionnaire development

Starting from the conceptual model, a logical, systematic and structured development process was undertaken, which included the following eight steps entailing qualitative as well as quantitative methods:

### Item generation

A Delphi procedure, which is a group facilitation technique including an iterative multistage process designed to transform opinion into group consensus [24], was applied to generate items to measure each of the 12 cells of the matrix. Hence, through successive Delphi rounds items were gathered, refined and synthesized by ranking and prioritizing opinions to shape the initial version of the HLS-EU-Q. In the first Delphi round all nine research teams in the HLS-EU Consortium were invited to propose items related to the twelve sub-domains in the HLS-EU matrix. The second Delphi exercise dealt with the selection of items proposed in the first round. The Delphi rounds were conducted through email to permit efficient participation and respondents had opportunities to revise their inputs and comment on summary feedback at each stage.

### Focus groups

To test the face validity of the draft questionnaire, focus groups were organised by the HLS-EU project partners in Ireland (JF, GD), Greece (BK, DA) and the Netherlands (VS, KS, SvdB). Participants provided informed consent and each group were invited to give feedback on the design, clarity and content of the questionnaire. To keep the costs low recruitment of participants for the focus groups was done via convenience sampling [25], which entailed involving the most accessible, participants with knowledge on health and preferably health literacy as well as general citizen skills. Hence, the sample included students and academic staff from the three participating universities, respectively. The characteristics of the participants are outlined in Table 2.

**Table 2:** The characteristics of the focus group sample

**Characteristics**	**Greece**	**Ireland**	**The Netherlands**
**Number of participants**	Eight	Five	Six
**Age**	22-64	24 – 47	20 -28
**Gender**	2 men/6 women	3 men/2 women	2 men/4 women
**Nationality**	Greek (5)	Irish	German
	Greek/American		Maltese
			Dutch
**Profile**	Sociologists (2)	Developmental economist;	Students in the Bachelor and Master Programme of European Public Health
	Philosophy and semiotics	Sociologist with MSc. Urban	
	Health economics	planning;	
	Social worker and public	Sociologist;	
	health specialist	Trainee accountant with background in accountancy and tax law	
	Journalist and lawyer		
	Elementary teacher		
	Administrative worker		

### Pre-testing

The revised version resulting from the focus-group feedback was field-tested in two countries (Ireland and the Netherlands). The field test included 50 computer assisted face-to-face interviews in each country conducted by JF in Ireland and VS and KS in the Netherlands. To recruit participants for this field test, judgement sampling, also known as purposeful sampling was used to guarantee an equal distribution of participants in terms of the parameters age, gender and education [25]. Due to an incorrect saving procedure data from one Dutch interview was lost, leaving a total of 99 interviews. The interview time varied from 25-90 minutes. The profile of the sample is described in Table 3.

**Table 3:** The characteristics of the pre-test sample

Gender	42 males
	57 females
Age	15-81 years old
	(mean 43.8)
Education	24% no formal education or primary education
	34% secondary education
	9% vocational training
	32% tertiary education
Employment	62% working
	15% studying
	23% not working
Health-related employment	83% never worked in the health-care sector
	27% worked or had worked in the health care sector

The methodological approach concerning data analysis involved both a qualitative and quantitative analysis of the data. For the qualitative analysis, data derived from logbooks and observations made by the interviewers and general comments and feedback from participants were scrutinized using the recommendations to refine the questionnaire. The quantitative analysis involved an item analysis, Principal Component analysis (PCA) and reliability analysis on the scores of the respondents on the questionnaire items. For the item analysis, the distribution of the responses on each item was inspected to eliminate items with a low discriminative power (i.e., 95% or more of the answers in the same category). For the PCA, a separate analysis was performed for each domain (healthcare, disease prevention and health promotion), with the number of components fixed at four related to the four information-processing dimensions outlined in the health literacy matrix derived from the conceptual model and definitions and a VARIMAX rotation to yield maximum discrimination between the components. The resulting factor structures were inspected, and items without sufficient loading (< 0.30) on any of the components or with a small difference in factor loading on any two components were excluded. The remaining items were again entered into PCA. This iterative procedure was repeated until an interpretable component solution was obtained. Subscales were constructed on the basis the highest component loading of an item. The internal consistency of the scales obtained through the PCA was tested by means of the Cronbach’s alpha.

### Expert consultation

In addition to the field test, consultations were organised with experts in the field of health and health literacy to assess the construct validity as well as the technical qualities (scaling, ordering of items etc.) of the questionnaire. The experts were recruited from the national advisory panels that had been established as part of the HLS-EU Project and among the collaborative partners of the HLS-EU project to gain second opinions supplementing the work carried out by the HLS-EU Consortium [22,26].

### Finalisation of the questionnaire

The results of the pre-test, field test and expert consultations, respectively, were pooled and evaluated by a sub-group within the HLS-EU consortium (KS, JP, SvdB, ZS, GD) supplemented with input from the collaborative partner (RO). Items that did not fit well within the conceptual model and rationale of the questionnaire or which did not have direct or indirect relevance to the twelve sub-domains were eliminated. Items that were only indirectly associated to the rationale of the questionnaire were combined with other items. Proposed objective items such as questions related to concrete knowledge were discharged due to cultural discrepancies among the participating countries. Instead, it was decided only to include self-reporting items, similar to the practice of Chew et al. [27]. Hence, the format of all items was changed from ‘statements’ to ‘questions’, and their formulation standardized so that all would assess the difficulty of a specific health relevant task, i.e.: “On a scale from very difficult to very easy, how easy would you say it is to …followed by the question to be answered on a Likert-type scale ranging from “very easy”, “easy”, “difficult” or “very difficult”. An answer category was added as “I don’t know”, which was only to be used by the interviewer. While it was ensured that the reformulated items stayed true to the original content, some new items were added, although not tested, to replace items that had been eliminated during the ‘culling’. This procedure resulted in a pre-final version of the questionnaire

### Plain language check

The pre-final questionnaire was examined for plain language by literacy experts from the National Adult Literacy Agency in Ireland for its compliance with plain language guidelines.

### Translation

The English version of the final questionnaire served as a master version for translation into the six other languages to be used in the European Health Literacy Survey (Bulgarian, Dutch, German, Greek, Polish and Spanish). Two independent translators translated the questionnaire from English, to the target language. For each language, a panel consisting of national research partners, the European Health Literacy Survey Coordinator, the translators and other relevant health professionals assessed the two translations with the aim of agreeing to a formal national version of the HLS-EU-Q.

For each step, protocols were made to ensure standardization of the procedures across the countries involved.

## Results

The results of each of the eight development steps are described in the following and the items included in the final questionnaire are presented in the Additional file 1.

### Item generation

According to the two successive Delphi rounds, the first Delphi resulted in a total of 136 generated items across the 12 sub-domains of health literacy. Most of the items were self-report statements, to be answered on a five-point Likert scale. In addition, two to four items in the form of objective knowledge tests were formulated for each domain (healthcare, disease prevention and health promotion) to test the level of achieved health literacy. The second Delphi dealt with the selection of items proposed in the first round, and resulted in a reduction to 43 health literacy items across the 12 sub-domains. This round was based on partial consensus. The final decisions were made by the coordination team from Maastricht University [KS, SvdB and VS] in the process of creating the draft version of the questionnaire to be tested in focus groups.

### Focus groups

The focus group discussions resulted in feedback on the structure, clarity and content of the questionnaire.

With regard to the *structure*, participants suggested to change the order of items from focusing on the competencies related to information processing to a focus on the domains of healthcare, disease prevention and health promotion. Another comment with regard to the structure of the questionnaire concerned its repetitiveness. With regard to *clarity*, it was mentioned that the questionnaire was expert biased. Finally, with regard to *content*, remarks were that (i) the ‘objective’ questions and the ‘knowledge’ questions, were too difficult; (ii) items might be culturally sensitive, as some questions were not generic enough to cover differences in health systems and contexts across the eight countries; and (iii) some items were found to prompt socially/culturally acceptable answers, e.g., related to health beliefs. In addition, there were concerns about privacy and the extent to which respondents would be willing to share their opinion and reply to the health literacy related questions and, especially, to socio-economic status related questions.

Following the incorporation of the comments and suggestions from the focus groups, a revised version of the questionnaire was made, which consisted of 47 items associated to health literacy: 22 for the healthcare domain (of which 19 self-report items and 3 objective items testing the level of achievement); 13 for the disease prevention domain (10 self-report and 3 test items); and 11 for the health promotion domain (9 self-report and 2 test items).

### Field test

The *qualitative analysis* of field test data derived from logbooks, observations by the interviewers and general comments from both the Irish and Dutch participants pointed out that although the content of the questions was well-understood and considered as appropriate, the questionnaire was seen as too lengthy, too comprehensive, too repetitive, and too expert biased in terms of language (e.g., use of the word “hypertension” instead of “blood pressure”). It was also considered very time-consuming, with the interview time varying between 30 and 90 minutes, with an average of approximately 60 minutes. As a result of these comments, the design of the questionnaire was slightly changed after the first 10 interviews. Highly similar items were combined into sets of questions, to avoid repetition and facilitate the process of answering the questions.

With regard to the quantitative analysis of the data from the field test (N = 99), the distribution of the responses to the items indicated that all items except from two showed sufficient variation across the response categories (i.e., less than 95% of responses on a single category). These two items were discarded. The PCA on the self-reported items measuring health literacy in healthcare, after three iterations, resulted in a four-component solution explaining 59% of the common variance. The PCA on the self-reported items concerning disease prevention, after four iterations, resulted in a four-component solution explaining 64% of the common variance, and finally the PCA concerning health promotion, after four iterations, resulted in a four-component solution explaining 62% of the common variance. In conclusion, these results indicate that for each of the three domains (healthcare, disease prevention and health promotion) a four-component structure was found which reflected the four dimensions of accessing, understanding, appraising and applying health related information. Alpha Cronbach levels ranged between 0.51 and 0.91. Taking into account that Cronbach’s alpha is sensitive to a low number of items, these values suggest that the obtained scales are reasonably homogenous.

### Expert consultation

The expert consultations (N = 25) requiring second opinions from members of the health literacy advisory boards and other collaborative partners in the HLS-EU project resulted in a series of critical reflections about the content and format of the questionnaire. The main recommendations by the experts were to: (1) remain true to the aim of the questionnaire (i.e. measuring health literacy in the general population) by looking at people’s competencies to access, understand, appraise and apply information to take decisions in terms of disease management, risk management and health management; (2) keep the focus on people, patients and lay persons; the system efforts are not the main focus of this survey; (3) maintain a generic approach, recognizing that health literacy is content and context specific and that the survey will be applied across many countries and different cultural settings; (4) keep the design simple at all levels – from lay-out to content such as items and response categories; (5) ensure clear language and avoid “expert” terminology; (6) keep the questionnaire easy to administer.

### Finalisation of the questionnaire

The final item selection process integrating the results of the focus groups, qualitative and quantitative analysis of the field test data, and expert consultations, yielded a pre-final version of the HLS-EU-Q which differed from the field tested version in several ways. While still closely related to the conceptual model and matrix, it was less repetitive; it included plain language and contained only self-reported items. The intended ‘objective’ items such as the knowledge questions were discarded due to lack of consistency across the eight countries. Literacy items related to e.g. word recognition and text comprehension were also not included. The core questionnaire contained 47 health literacy related items, covering the 12 sub-scales of the HLS-EU matrix with 3-5 items in each scale. The number of items in each scale was a result of the consensus-based item selection process within the sub-group. The exact wording of each item is presented in the Additional file 1.

### Examination for use of plain language

The examination of the questionnaire by the National Adult Literacy Agency in Ireland (NALA) resulted in a number of smaller changes to accomplish more simple language in the final version of the questionnaire e.g. “…judge the reliability of illness-related information presented in the media?” was changed to “…judge if the information about illness in the media is reliable”. The review by NALA ensured that the items were easy to read and understand which in turn facilitated that data collection would run more smoothly and quickly, than was experienced in the pre-test and field-test.

### Translation

The translation of the final version of the questionnaire produced identical versions of the questionnaire in 7 languages ((Bulgarian, Dutch, English, German, Greek, Polish and Spanish). In addition, the English version was adapted in its original version to be applied in Ireland, and the German version was adapted for use in Germany and Austria by the translation panel and the experts involved in the respective countries to ensure its cultural applicability e.g. in terms of translations of specific words that differed or system-related items.

An overview of the characteristics of the design and development process for the HLS-EU-Q is presented in Table 4.

**Table 4:** The characteristics of the design and development of HLS-EU-Q

**Purpose**	The rationale for the HLS-EU-Q is to address the lack of European data on health literacy in populations by providing an adequate instrument for collection of data, which can generate insights on national perspectives as well as a comparative analysis of the state of the art of health literacy in Europe.
**Research question**	The aim of the questionnaire is to measure health literacy in (European) populations with reference to the HLS-EU definition and conceptual model on health literacy as outlined by Sorensen et al. [23]
**Scale and response format**	Likert-type scales with a four choice format “Very easy, easy, difficult, very difficult”; “Don’t know” only to be ticked by interviewer.
**Generation of items**	Items generated by a Delphi procedure among consortium members, expert consultation, and literature review with reference to the HLS-EU conceptual model and a deducted matrix suggesting 12 sub-domains of health literacy.
	47 core items were generated, placed first in order of the four information processing dimensions (accessing, understanding, appraising and applying health information to take decisions), then changed to be ordered in relation to the three health domains in focus: healthcare, disease prevention and health promotion. Within these sub-domains, items were placed in logic order according to content and purpose.
	A subsequent second section focused on antecedents and consequences of health literacy related to the HLS-EU conceptual model operationalized as 39 items on personal information; health service use, health behaviour, community participation and socio-economic factors.
**Test and pilot of items**	Pre-test concerning face validity was made in three focus groups in Greece, Ireland and the Netherlands, respectively.
	Field test was conducted as face-to-face interviews in Ireland (n = 50) and in the Netherlands (n = 49) to measure quantitative and qualitative aspects of the measurement.
**Amendments based on item analysis or related techniques**	Amendments were made based on
	- pre-test
	- field test
	- consultation process
	- plain language examination
	- translations
**HLS-EU-Q versions**	HLS-EU-Q47 (core health literacy related items only); HLS-EU-Q86 (measuring health literacy as well as antecedents and consequences according to the HLS-EU conceptual model).
**Creation of an independent data set**	The HLS-EU-Q86 was applied as part of the European Health Literacy Survey (HLS-EU) in a sample of 8000 participants from the general populations in Austria, Bulgaria, Germany, Ireland, the Netherlands, Poland and Spain [28].

## Discussion

Health literacy has gained increasing attention in research and practice over the past decades [29]. With this increasing interest, there has been a growing demand for tools to measure health literacy [30]. However, existing tools to measure health literacy focus mostly on screening for functional health literacy within clinical settings by e.g. testing word recognition or the understanding of food labels [11,31]. As HLS-EU-Q was developed for measuring the health literacy of general populations and not of specific patient groups, it does not follow a narrow clinical or medical focus, but captures a broad public health perspective. Grounded in public health, the HLS-EU-Q measures health literacy in terms of three domains where people’s health is of concern and is expressed in terms of accessing, understanding, appraising and applying information to manage disease, manage risks and manage health. In other words as a patient being ill navigating the healthcare system, as a person at risk encountering information on disease prevention, and as a citizen striving for optimal health encountering health promotion offers in the community, the work place, the educational system and the market place as described in detail by Sorensen et al. [23] As illustrated in the HLS-EU model the assumption is that health literacy is the outcome of informal and formal learning and health education. This means health literacy refers to an evolving set of competencies that do not remain static over time. In this definition, health is linked to quality of life and can be regarded as a means to an end rather than a fixed state, to which a person should aspire: “(Health is) the extent to which an individual or group is able on the one hand, to realize aspirations and satisfy needs; and, on the other hand, to change or cope with the environment. Health is, therefore, seen as a resource for everyday life, not an object of living; it is a positive concept emphasizing social and personal resources, as well as physical capacities” [32].

### Bridging measurement gaps

The HLS-EU-Q addresses many of the shortcomings of existing tools brought forward by e.g. Jordan et al. [20] and Pleasant et al. [6]. It is explicit build on a definition and a conceptual framework of health literacy. It is multi-dimensional in content and distinguishes health literacy from communication. It treats health literacy as a ‘latent construct’ and follows a principle of compatibility, since different scales can be used for different contexts. It permits comparison in different populations and makes reference to public health rather than just clinical use. The development process of the measurement instrument has been based on a consensus approach involving nine research teams from eight countries as well as a large number of collaborating partners from Europe and abroad, all with a variety of professional educational backgrounds and experiences. Though the cross-national and cross-disciplinary group have been an advantage to ensure a wide range of perspectives in every step of the development process, the multifaceted group also remained a challenge throughout the process since the overall initial demands from all the investigators involved to content and form turned out to be too comprehensive and lengthy and therefore not feasible to administer. By respecting the feedback from users, participants and external stakeholders crucial decisions were taken during the development process, which considerably impacted the design of the final version. Eventually, the new orientation and the accompanying process of scrutinizing every item for its suitability according to agreed quality criteria paved the way for a stringent tool, matching the aim of the study while still staying true to the original ideas presented in the definition, concept and questionnaire matrix of the HLS-EU Consortium. The variation in interview time depended on the responses made by the participants as some were fast and could answer quickly, while others took longer because they found it difficult to deal and manage their health and had to reflect a longer time. The choice of a 4 point-Likert scale differentiated the responses, and for those who wished to skip an answer or could not answer items, the interviewer had the option of the “I don’t know” category. Due to the fine-tuning of the questionnaire the administration time was reduced to an average of 20-30 minutes including an additional section on personal back-ground variables referring to e.g. demographic and socio-economic factors that was also included in the test phases.

### Limitations

Several limitations should be noted in the design and development of the HLS-EU-Q. The Delphi generated items mainly in the domains of healthcare and disease prevention and less in the domain of health promotion. Furthermore, the Delphi resulted in an excess of items, hence the process resulted in partial consensus where the coordination team adjusted the questionnaire by e.g. selecting the most preferred items. In addition, there was limited geographical scope applied in the testing phase. Focus groups were only carried out in three countries and the field test applying face-to-face interviews only in two. Ideally, it would have been better to include all countries, but this was not feasible within the financial constraints of the study. The data analysis regarding the field test revealed a variation of Chronbach’s alpa from 0.51 to 0.91, which warrants further research, since some of the values were considerably low. The overall results of the focus groups, the field test and the expert consultations generated a change in the item design, hence it is suggested that the aspect of Chronbach Alpha is taken specifically into consideration when validating the questionnaire in its application e.g. in the European Health Literacy Survey. Although, the expert consultations involved experts from Ireland, Israel and Australia (see acknowledgement) adding to the multi-national perspectives already represented in the HLS-EU Consortium, an even wider representation may have been beneficial for the questionnaire’s applicability in different cultures. The continuous feedback throughout the development process on problems concerning expert biases in terms of difficult words and wordings emphasized how difficult it was for the wider group of researchers to let go of expert language and underlying paradigms. In response, a plain language check was carried out by a designated Literacy Agency to ensure a final assessment of clear and transparent language in the questionnaire. The translations involved professional translators only in the final step after the plain language check. Ideally professional translations would have been carried out for the pre-test and field test as well. By illuminating the overall research process, as well as the detailed steps in the design and development process undertaken by the HLS-EU Consortium, the necessary transparency is offered for others to apply the HLS-EU-Q with a deeper understanding of its purpose, its capability and its limitations. Yet, it is also evident that further research is needed to enhance its quality and applicability in the future.

## Conclusion

This paper has explained the design and development process of the European Health Literacy Questionnaire, the HLS-EU-Q. By illuminating the detailed steps in the design and development process of the HLS-EU-Q, a deeper understanding of its purpose, its capability and its limitations has been provided for others using the tool. Bearing these insights in mind it is the vision that HLS-EU-Q with its conceptual-based, multi-facetted attributes will be validated in more countries to enhance the understanding of health literacy at population level.

## Endnote

^a^While in most participating countries, samples representative for the whole country were sampled, however for feasibility reasons in Germany only the biggest state North Rhine Westphalia took part in the study!

## Competing interests

The authors declare not to have any competing interests.

## Authors’ contributions

Background: KS; SVdB; JMP; GD; ZS; BK; HB. Method: KS (Delphi, focus groups, field-test, expert consultations, translations); SVdB (Delphi; field test, expert consultations); JMP (Delphi, expert consultations, translations), JF (Delphi, focus groups, field test, expert consultations); GD (Delphi, focus groups, field test, expert consultations); ZS (Delphi, expert consultations, translations); BK (Delphi, focus groups, expert consultations, translations); RHO (expert consultations), HB (expert consultations). Results: KS; SVdB; JMP; JF; GD; ZS; BK; RHO; HB (collaborative development of the HLS-EU-Q during seminars and telephone conferences on the basis of outcomes of the design and development steps). Discussion: KS; JMP; GD; ZS; BK; RHO; HB. All authors read and approved the final manuscript.

## Pre-publication history

The pre-publication history for this paper can be accessed here:

http://www.biomedcentral.com/1471-2458/13/948/prepub

## Supplementary Material

Additional file 1 Annex. The HLS-EU-Q47 of the HLS-EU Consortium applied in the European Health Literacy Survey (HLS-EU). Click here for file
